# Investigating the intention of purchasing private pension scheme based on an integrated FBM-UTAUT model: The case of China

**DOI:** 10.3389/fpsyg.2023.1136351

**Published:** 2023-03-09

**Authors:** Guo Wu, Jiaying Gong

**Affiliations:** ^1^Department of Finance, Shengxiang Business School, Sanda University, Shanghai, China; ^2^Business School, Cardiff University, Cardiff, United Kingdom

**Keywords:** behavioral intention, UTAUT, Fogg Behavior Model, private pension scheme, structural equation modeling

## Abstract

The newly established private pension scheme in China has received great attention as it would be an important supplement to China’s social safety net and corporate annuity amid an aging population. It provides a way of helping to address the challenge of ensuring adequate retirement income, and the scheme is expected to grow significantly in the coming years. This study investigates factors affecting the intention of purchasing the private pension scheme using a conceptual model based on the integration of Fogg Behavioral Model (FBM) and Unified Theory of Acceptance and Use of Technology (UTAUT) model. The questionnaire-based data from a sample of 462 respondents had been analyzed. Both exploratory factor analysis and confirmatory factor analysis were used to assess validity. The hypothesized relationships in the integrated FBM-UTAUT model were tested using structural equation modeling. The research findings indicate that anticipation, social influence, effort expectancy, performance expectancy, side benefits and facilitating conditions have significant positive impacts on intention to purchase. According to the exploratory factor analysis, the integrated FBM-UTAUT model can explain more than 70% of the total variance. Meanwhile, effort expectancy can be affected by time effort, thought effort and physical effort collectively, while performance expectancy can be affected by risk and trust. It is revealed that the integrated FBM-UTAUT model can be effective in explaining purchase intentions in a private pension scheme context, and this study is expected to offer helpful advice on the design of pension products and the reform of pension policies.

## 1. Introduction

### 1.1. China’s private pension scheme

The third pillar of China’s pension system, also known as the private pension scheme, was officially launched on November 25, 2022. China is facing challenges in coping with the expansion of its aging population and the declining birth rate, the new private pension scheme has therefore been regarded as a significant supplement to the existing two pillars, social safety net and corporate annuity (Feldstein, 1999; Leckie and Pan, 2007; Liu, 2022). First, individual income taxes contribute to the state-sponsored pension (the first pillar) in part, but as the number of people in working age declines, this source of funding will be reduced. According to a study by the Chinese Academy of Social Sciences, the social pension fund is anticipated to experience a deficit of more than 100 billion RMB by the end of 2030 (Cai and Cheng, 2015). Second, the employer-sponsored pension plan (the second pillar) only covers less than 30 million people in China. Most small and medium-sized enterprises (SMEs) are unable to provide corporate annuities because of high operating costs, and many of them even fail to pay the required social security for their employees (Dong and Wang, 2016). As a result, the third pillar of private pensions is urgently required to ensure the financial security and stability of China’s aging population. Making sure that people will continue to have access to disposable income after retirement is also in the best interest of the country as a whole.

According to the notice published by China’s Ministry of Human Resources & Social Security, people in 36 cities can start opening accounts for the new pension scheme. People enrolled in China’s private pension scheme can voluntarily contribute up to 12,000 RMB each year to their individual retirement accounts, which are tax-deductible. Participants can utilize their pension contributions to buy financial products from approved financial institutions after setting up their individual retirement accounts. This has actually provided the financial industry with unprecedented opportunities in the commercial pension business. Relevant financial institutions, such as banks, insurance companies, and mutual funds, must take societal demands into consideration, increase the availability of financial products and services, and support the sustainable growth of China’s multi-pillar pension system.

The research objectives of the study are: first, to propose a new conceptual model to analyze the factors that potentially influence people’s intention to purchase the new private pension scheme based on the Fogg Behavior Model (FBM) and the Unified Theory of Acceptance and Use of Technology (UTAUT) theory; second, to evaluate the robustness of an integrated model and to test hypothesized relationships between factors using a structural equation modeling approach; third, to provide helpful advice in terms of product design and pension policies according to the research findings. The present study is expected to bridge the knowledge gap in two aspects. To begin with, most existing studies regarding China’s pension schemes have focused on aspects related to pension product design (Chen, 2016; Chen and Turner, 2021), institutional arrangements (Salditt et al., 2008; Shi and Mok, 2012), investment strategies (Leckie and Pai, 2005; Zhang and He, 2009; Shen et al., 2020), tax deferral policies (Dong and Wang, 2016; Liu and Sun, 2016; Li, 2020) and the sustainability of pension systems (Zhao and Mi, 2019). There are currently no published studies that aim to systematically analyze China’s private pension scheme from a psychological perspective. The mechanisms that underpin people’s behaviors are not fully understood in the context of private pension scheme. The study is expected to provide a valuable reference for pension product designers and policymakers. Moreover, it is known from the literature that the factors (such as effort expectancy, performance expectancy, social influence, and facilitating conditions) in the classic UTAUT model have been shown to be significant in predicting behavioral intentions (Malik, 2020). The UTAUT model has received great attention in technology-related contexts (Williams et al., 2015). Despite its popularity, there still remains a paucity of empirical evidence in other contexts. Some recent studies have explored its suitability in finance-related contexts (Bommer et al., 2022; Firmansyah et al., 2023). The present study is hoped to further explore the UTAUT constructs in a private pension scheme context with the integration of other new factors.

In this paper, we propose an integrated model based on the combination of the FBM and the classic UTAUT model. The FBM highlights three principal components, including motivation, ability, and trigger (Fogg, 2009). The three components collectively determine whether a behavior occurs or not. The FBM framework has been favored by researchers in assessing behavior change, and the organized structure has been adopted in a variety of contexts recently (Agha et al., 2019; He et al., 2019; Du et al., 2022). We therefore see its potential merit in explaining the behavioral intention. Meanwhile, the UTAUT is built on a variety of theories, such as theory of reasoned action (Ajzen and Fishbein, 1977), theory of planned behavior (Ajzen, 1991), social cognitive theory (Bandura, 2001), and diffusion of innovation theory (Rogers et al., 2014). The UTAUT has been extensively and successfully applied in different technology-related contexts (Williams et al., 2015; Khechine et al., 2016; Taherdoost, 2018), and various cultural settings have also been reported (Im et al., 2011; Malik, 2020). We find that there has been a growing trend of its extension on finance-related contexts recently (Yu, 2012; Bhatiasevi, 2016; Jiang et al., 2019; Xie et al., 2021), and some UTAUT constructs could exhibit sufficient predictive power (Venkatesh et al., 2011; Chang, 2012). Therefore, in respect of the current research context, we propose an integrated FBM-UTAUT model with the incorporation of some other variables that could improve the overall reliability of the conceptual model.

The rest of the paper is structured as follows: section 1.2 reviews related literature and introduces the theoretical foundation regarding the FBM-UTAUT model; section 2 shows the development of hypothesized relationships; section 3 introduces the research methodology, including survey design, sampling, data analysis and demographics; section 4 presents the results related to the test of reliability, the assessment of validity, and the path analysis *via* structural equation modeling; section 5 discusses the economic significance as well as the practical implications of the research findings; second 6 finally provides concluding remarks.

### 1.2. Supporting theories

This section reviews the supporting theories beneath the integrated FBM-UTAUT model. The Fogg Behavior Model (FBM) has been proposed as a model for comprehending human behavior (Fogg, 2009). According to this model, motivation (a person is sufficiently motivated), ability (a person has the capability of performing a behavior), and trigger (a person is triggered) are the three aspects that determine the behavior. In other words, when all three of the preceding conditions are met, an individual will engage in a behavior. Researchers (Fogg, 2009; Ferebee, 2010; de Toledo et al., 2018; Meekers et al., 2020; Mota et al., 2020) have applied the FBM model primarily in fields related to persuasive technologies. This field of research can be described as studies regarding the technologies that act as agents in behavioral persuasion and the change of users’ attitudes. Guimaraes et al. (2018) presented a generalized mathematical modeling for the Fogg Behavior Model and showed its effectiveness in evaluating persuasive technologies. Unfortunately, there is little work reported in other contexts. Providing its straightforwardness, it is also academically interesting to shed some light on its effectiveness in different fields. At least, the FBM can lay a fundamental framework to start with. In the meantime, although the FBM is very qualitatively straightforward for understanding behavior occurrence and behavior change, there are still insufficient studies on how motivation, ability, and triggers can be accurately measured by any specific variables. To bridge this gap, we use several variables to reflect the three key dimensions of the FBM in a more deterministic way.

The Unified Theory of Acceptance and Use of Technology (UTAUT) is perhaps the most widely used theory for studying technology acceptance and usage adoption in organizations (Venkatesh et al., 2003; Rahman et al., 2017). The UTAUT contains four critical variables, including effort expectancy (EE), performance expectancy (PE), social influence (SI) and facilitating conditions (FC). According to Venkatesh et al. (2003), the UTAUT could explain up to 70% of the variance in users’ behavior regarding technology adoption. The extant literature has indicated that the UTAUT is a reliable theoretical model to investigate the factors affecting human behavior in various contextual settings (Chang, 2012; Williams et al., 2015; Dwivedi et al., 2020) and cultural settings (Im et al., 2011; Al-Qeisi et al., 2015; Abbad, 2021). We also find that the UTAUT model has been applied in a few finance-related contexts. For example, Cheng et al. (2009) established a model on the basis of the UTAUT model to predict user acceptance of internet banking. Yu (2012) used the UTAUT model to examine the factors influencing people’s intentions to use mobile banking from a sample of 441 respondents. The author showed that the four core variables of the UTAUT model could significantly impact behavioral intention. Bhatiasevi (2016) later extended the UTAUT model and incorporated the perceived financial cost variable to explain the adoption of mobile banking, and the result was similar. Lee (2013) used the UTAUT model with the addition of a security risk variable to study the determinants of intention to buy online automobile insurance products from a sample of 203 respondents. Jiang et al. (2019) reported an investigation of online life insurance purchase intention, and the authors successfully applied the UTAUT model as a foundation for the conceptual model. As can be seen from the above discussion, the core constructs in the UTAUT model can be quite robust in predicting behavioral intention. Unfortunately, there is still a scarcity of literature on the UTAUT model in non-technology contexts, and it is encouraged but also academically interesting to pay more attention to its adaptability. We see the potential capability in forecasting purchase intention using the UTAUT constructs in a private pension scheme context.

As such, this paper reports an investigation of the behavioral intention to purchase the private pension scheme using variables from both the FBM and the UTAUT, resulting in an integrated FBM-UTAUT model. We also incorporate antecedent factors that may significantly determine effort expectance and performance expectance. Section 2 presents the development of hypothesized relationships between these variables.

## 2. Hypothesis development

### 2.1. Dependent variable: Intention to purchase

Intention to purchase can be viewed as a concept that reflects the psychological willingness of a person to make purchasing decisions in a certain organization (Morrison, 1979). It is often thought to be a result of customers’ attitudes, evaluations, and other decisive factors (Chang and Wildt, 1994; Akar and Nasir, 2015). More broadly speaking, purchase intention can be seen as the intention to perform a specific behavior that would eventually result in a buying behavior. Therefore, some researchers (Jansen et al., 2007; Kathuria et al., 2010) classify purchase intents into four types: informational intent, investigative intent, navigational intent, and transaction intent. The transaction intent is the one that is most equivalent to actual purchase intent and conversion. We define intention to purchase in this study as a degree of an individual’s propensity to purchase the private pension scheme.

Researchers have proposed different kinds of theories to elucidate the decision-making process of customers. For example, the theory of reasoned action (TRA) was first proposed by Ajzen and Fishbein (1977) and it describes the relationships between a person’s perception, norms, and attitudes. These factors can collectively predict the intentions of a person in making decisions. Bandura (1989) later developed the social cognitive theory (SCT), which posits that environmental factors, personal factors, and behaviors are determined reciprocally. Davis (1989) adapted the TRA and proposed the technology acceptance model (TAM), which consists of two critical factors (perceived usefulness and perceived ease of use). In contrast with TRA, Davis et al. (1989) dropped subjective norms due to weak psychometric results. Malhotra and Galletta (1999) further stated that social influence may not have a direct impact on behavioral intention. Ajzen (1991) further extended the TRA and incorporated perceived behavioral control, this resulted in the theory of planned behavior (TPB). Rogers (1995) later proposed the innovation diffusion theory (IDT) to describe the factors affecting the acceptance of innovations. Venkatesh et al. (2003) combined multiple theories and developed a unified theory in which four main factors can have significant impacts on usage and intention. The four factors are: performance expectancy, effort expectancy, social influence, and facilitating conditions. It has been shown that the UTAUT model has the capability to justify around 70% of the variance in behavioral intention, comparing to 40% in other models (Venkatesh et al., 2003, 2011; Alshahrani and Walker, 2017). The UTAUT model has therefore received great attention, and it presents a basis to guide the research in usage behavior study. This paper also considers factors in the UTAUT model to build the conceptual framework.

**Figure 1:**
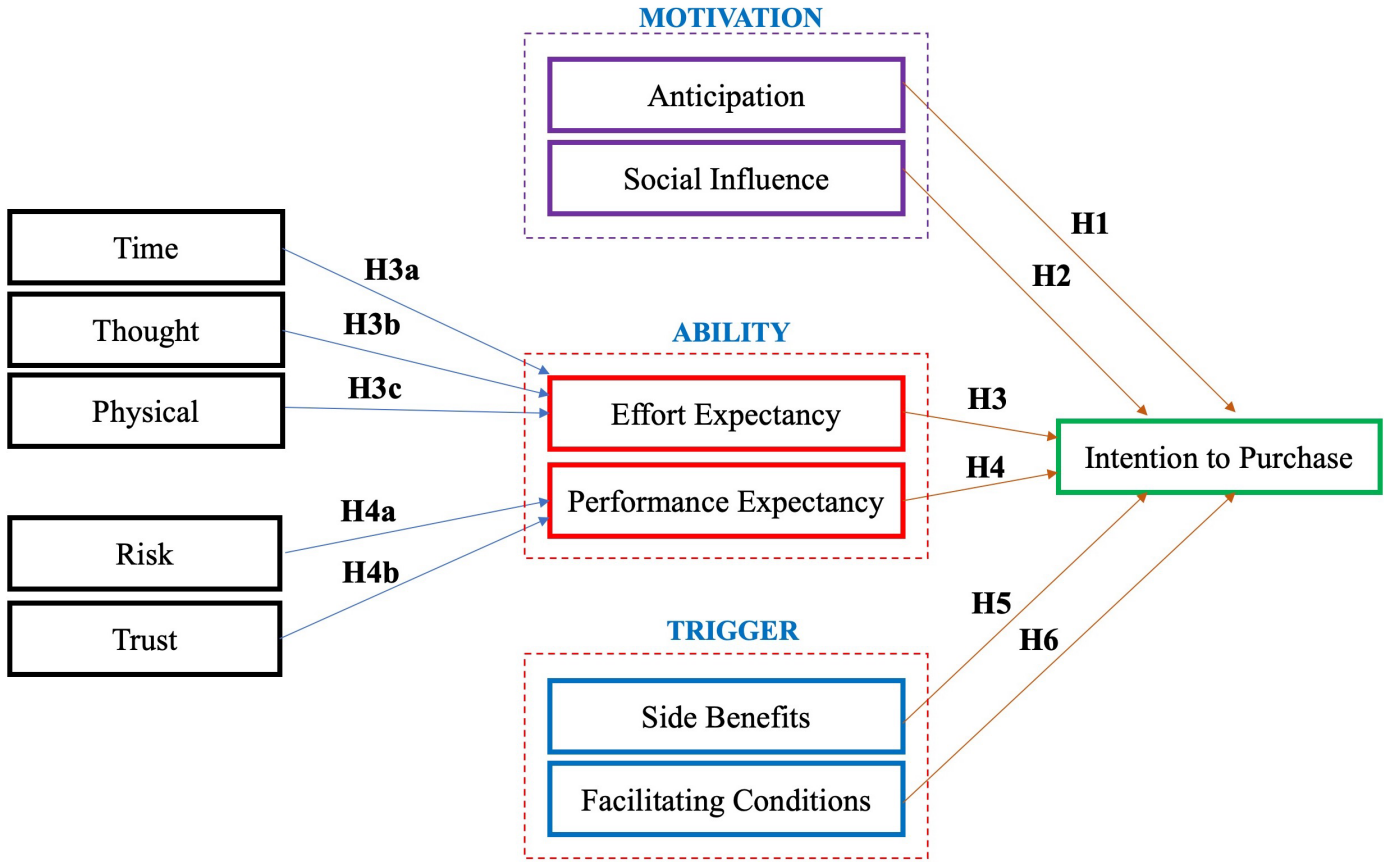
The conceptual model.

According to the extant literature, intention to purchase is in fact complex and can be affected by a variety of factors simultaneously. This article specifically explores the critical factors driving the purchase intention of China’s private pension scheme on the basis of an integrated FBM-UTAUT model. The conceptual framework is summarized in Figure 1. The proposed model consists of multiple dimensions. The following sections will describe the variables associated with these dimensions in details and also illustrate how they impact purchase intention hypothetically.

### 2.2. Motivation dimension

According to the FBM, motivation is the first key element, and it describes the underlying drive that motivates a person to perform a certain behavior. A higher level of motivation likely induces a higher level of behavioral intention. In the FBM-UTAUT model, we introduce anticipation (a factor mentioned in the FBM) and social influence (a variable used in the UTAUT) to collectively reflect the overall motivation of a person’s intention to purchase the private pension scheme.

First, anticipation refers to the emotional aspect of motivation and captures a person’s hope and desire. This factor has drawn researchers’ attention recently in public health related contexts. Alrige et al. (2021) reckoned that anticipation is the most critical motivator in a precautionary health behavior study during the COVID-19 pandemic, while Agha (2022) reported a behavior study on COVID-19 vaccination. The researchers discovered that people with a higher anticipation are more likely to adopt a behavior. In this study, anticipation is a variable that describes the degree to which people hope that the private pension scheme will help them meet investment requirements, achieve inflation-hedged protection of wealth, and maintain their living standards after retirement. There are various studies that have explored the effect of anticipation on customer behavior. For example, Koenig-Lewis and Palmer (2014) carried out a study on the effects of anticipatory emotions on service satisfaction and behavioral intention from a sample of 204 respondents in a service context. The authors discovered that anticipatory emotions generated by these respondents when considering a forthcoming event would influence their behavioral intentions and post-experience emotions. Polegato and Bjerke (2019) found that pre-experience anticipation can significantly predict post-experience customer satisfaction. Soyer and Dittrich (2021) found a positive correlation between anticipation and customer behavior in purchasing clothes. In fact, anticipation may be traced back to the concept of consumption vision (Phillips, 1996), which depicts that people can imagine themselves performing a specific behavior. Consumption vision is regarded as a self-constructed mental simulation of a person’s future consumption scenarios.

Second, social influence refers to the social aspect of motivation, and it is usually defined as the level of how much a person feels the importance that the other social members believe the person should adopt the new product or service (Venkatesh et al., 2003). This sense of belongingness could be a significant motivator, making people feel that they are a part of a wider social community. Srite and Karahanna (2006) showed that individuals from a higher collective culture are more likely to comply with the opinions of salient others. Venkatesh and Zhang (2010) posited that the effect of social influence is distinct in China due to its collective culture (Hofstede et al., 2010). Empirically, Zhou and Li (2014) discovered that social influence plays a dominant role in the continuance usage of mobile social network services, while Zhang et al. (2020) identified a strong effect of social influence on automated vehicle acceptance in China. We expect that social influence could also play a significant role in this case. Existing studies have clearly elucidated the significant positive relationship between social influence and behavioral intention (Chang, 2012; Williams et al., 2015; Dwivedi et al., 2020; Malik, 2020) in various settings. The effect of social influence on intention to purchase a certain product or service in finance-related contexts had also been reported in the literature. For example, according to the literature review presented by Utami et al. (2021), the positive relationship is mostly robust regarding the adoption of FinTech products. Jiang et al. (2019) revealed that social influence has a significant positive impact on intention to purchase online life insurance using a sample of 315 participants in China. Keat et al. (2020) reported the same result using a sample of 183 engineering students in Malaysia.

Based on the above discussion, the following hypotheses are therefore developed:

*H1:* Anticipation has a positive impact on intention to purchase.

*H2:* Social influence has a positive impact on intention to purchase.

### 2.3. Ability dimension

#### 2.3.1. Effort expectancy

Effort expectancy (EE) was officially proposed by Venkatesh et al. (2003), and it refers to the degree to which people can use a technology, a system, or an application easily. As shown in various studies (Kim and Hunter, 1993; Snead and Harrell, 1994; Gupta and Arora, 2020; Fedorko et al., 2021), the ease-of-use expectation can be a crucial predictor of behavioral intention. In an earlier study, Radner and Rothschild (1975) posited that effort is a finite resource and if users spent less effort on a particular thing, they would therefore be able to allocate more resources to other things. Davis (1989) then suggested that an easy-to-use system would generate a positive attitude in a user, and the user would be more inclined to use the system. Moore and Benbasat (1996) later proposed the concept of complexity to reflect a person’s belief about whether using a system is effortless. Venkatesh et al. (2003) eventually included the construct effort expectancy as a key predictor of behavioral intention in the UTAUT model. Since then, EE has received great attention in various behavior studies across a range of research contexts (Venkatesh et al., 2011; Chang, 2012). In particular, there are some recent studies aimed at investigating the effect of EE on the adoption of financial products and services. For example, Alalwan et al. (2018) reported a study on the adoption of internet banking, Senyo and Osabutey (2020) investigated the intention to use mobile money services, Chan et al. (2022) addressed the factors affecting the use of open banking. All these studies support the significant relationship between EE and behavioral intention. In this study, we also consider EE as a critical factor in the integrated FBM-UTAUT model, and effort expectancy describes the extent to which people believe that purchasing the private pension scheme will be easy and effortless. In the meantime, as the extant literature relevant to the determinants of EE is scarce and we feel that having an in-depth understanding of the EE construct is necessary, we further consider possible antecedents of EE. In light of the current context, it is proposed that EE may be explained by three factors collectively: time, thought, and physical. First, as Mantel and Kellaris (2003) suggested, if consumers think they do not require additional time to find the information they need from a product, they would be more likely to use it. Kruger et al. (2004) proposed an effort heuristic theory which illustrates that the quality of product is positively correlated with the perceived amount of effort invested and the amount of effort is reflected by the amount of production time. This also elicits a possible positive relationship between effort expectancy and time (TIME). Second, thought (THO) refers to a term that describes the amount of mental effort required to complete a specific task. The concept of mental effort was systematically investigated by Mulder (1986), and the author reckoned that mental effort has a relationship with information processing complexity with respect to a task. This construct may be rooted in the theory of mental workload (Kantowitz, 1987), and it implies that if a person feels difficult to understand the task and requires extra thought, the person is more unlikely to successfully complete the task with ease. In this context, it is proposed that the overall expected effort is influenced by the amount of thought required to comprehend the details of the private pension scheme. Third, we propose that the expected physical effort is also one of the components determining effort expectancy. Although there is no explicit definition of physical effort in the literature, physical effort generally refers to the physical activities required for completing a specific task. Studies have indicated that people who perceive the use of a technology as requiring more physical effort will likely reduce their intentions to use the technology (Davis, 1989; Oh et al., 2009; Teo, 2011; Yuen et al., 2019). In this regard, it is assumed that physical effort expectancy (PHY) is a key determinant of effort expectancy.

Overall, according to the above discussion, the following hypotheses are formulated:

*H3:* Effort expectancy has a positive impact on intention to purchase.

*H3a:* Time effort expectancy positively influences effort expectancy.

*H3b:* Thought effort expectancy positively influences effort expectancy.

*H3c:* Physical effort expectancy positively influences on effort expectancy.

#### 2.3.2. Performance expectancy

In this study, we also place the UTAUT construct performance expectancy (PE) into the ability dimension. PE is the extent to which a person believes that the private pension scheme will improve his overall retirement welfare. According to earlier research, people are more likely to embrace and keep using new technology if they see that it has benefits that outweigh its drawbacks (Venkatesh et al., 2003, 2012). Additionally, numerous research findings indicate that PE has a significant impact on people’s propensity to use certain products or services (Williams et al., 2015; Hoque and Sorwar, 2017; Chao, 2019; Nan et al., 2022; Tian and Wang, 2022). For example, Chao (2019) reported a study on the use of mobile learning on the basis of the UTAUT model, and the author showed that PE has a significantly positive impact on behavioral intention with a coefficient of 0.18. Nan et al. (2022) studied the intention of using face recognition payment and also found a positive effect of PE on behavioral intention with a coefficient of 0.27. Tian and Wang (2022) found that the intention to adopt autonomous vehicles could be indirectly influenced by performance expectancy through perceived value. On the other hand, the antecedents of PE are in fact less understood, and existing studies have clearly indicated that risk and trust are two important factors determining behavioral intentions in various finance-related contexts (Gu et al., 2009; Slade et al., 2015; Kaur and Arora, 2020; Nan et al., 2022). In this regard, we assume that both risk and trust have significant impacts on the expected overall performance of the private pension scheme. First, risk can be defined as a variable that describes the level of doubts about the reliability of the private pension scheme and the degree of fear regarding the default risk of scheme managers. Existing studies have incorporated perceived risk into the UTAUT model as a factor that considerably hinders behavioral intention in various contexts, but the specific influencing mechanism is less understood. In this study, we propose that a person’s perception of risk has an indirect impact on purchase intention through performance expectance. Second, trust depicts the extent to which a person believes that a product or a service is trustworthy, the personal information is not revealed to others or security features that are present to protect his or her details (Slade et al., 2015). According to Chawla and Joshi (2019), the perception of trust promotes the adoption of technology-related payment systems. Similarly, researchers (Lu et al., 2011; Karim et al., 2020) have revealed that technology can provide high-level security measures to safeguard customers’ data and online transactions, fostering their desire and improving their perceived trust to utilize internet-based payment devices. In an online insurance context, Luo et al. (2021) studied the influence of people’s trust beliefs on their purchase intentions. Similarly, in an Islamic insurance context, Poan et al. (2022) showed that trust has a significant effect on purchase intention based on a sample of 322 respondents in Indonesia.

From the above analysis, we therefore develop the following hypotheses:

*H4:* Performance expectancy has a positive impact on intention to purchase.

*H4a:* Risk negatively influences performance expectancy.

*H4b:* Trust positively influences performance expectancy.

### 2.4. Trigger dimension

First, side benefits regarding China’s private pension scheme are explained as a belief that the scheme will provide extra benefits, such as tax benefits and fee free close-end management. Extra benefits would increase people’s overall perceived benefits regarding the private pension scheme. There are various studies (Lee, 2009; Dzulkipli et al., 2017; Ettis and Haddad, 2019; Putritama, 2019; Weedige et al., 2019) that have clearly illustrated the positive correlation between perceived benefits and behavioral intention to use certain financial services. For example, Ettis and Haddad (2019) found that perceived benefits would influence people’s attitudes toward e-insurance products, and attitude is a prominent factor determining behavioral intention. Weedige et al. (2019) also found a positive correlation between perceived benefits and the intention to purchase personal insurance products from a sample of 300 respondents in Sri Lanka. In fact, the influence of benefits on user behavior could be rooted in the prospect theory (Edwards, 1996), which suggests that people place more value on perceived gains rather than perceived losses. This implies that when given two options that are equal, a person will likely choose the one with the highest prospective gains.

Second, the FBM supposes that a trigger or a facilitator will influence behavior along with motivation and ability. The concept of facilitating conditions was first proposed by Venkatesh et al. (2003). In this study, we define the concept of FC as the degree of support that people feel they would obtain during and after purchasing the private pension scheme. In other words, FC describes the extent to which people believe that the organizational and technical infrastructures associated with the private pension scheme are readily available. We regard facilitating conditions as facilitators and suppose that the stronger the FC is, the higher the purchase intention will be. The existing studies have clearly supported the positive correlation between FC and behavior intention in various finance-related contexts, such as mobile banking (Afshan and Sharif, 2016; Bhatiasevi, 2016), health insurance (Ndifon et al., 2020), car insurance (Milanović et al., 2020), etc.

Consequently, we therefore put forward the following hypotheses:

*H5:* Side benefits has a positive impact on intention to purchase.

*H6:* Facilitation conditions has a positive impact on intention to purchase.

## 3. Research methodology

### 3.1. Survey design

The research hypotheses were tested using a quantitative approach based on survey data. Researchers propose that at least three measured items are required in each construct (Fabrigar and Wegener, 2012; Hair et al., 2014). A summary of the statements associated with each scale is shown in Appendix Table A1. All of the items were assessed using five-point Likert scales (from “1 = strongly disagree” to “5 = strongly agree”). Both the Chinese version and the English version were provided to fit the needs of all respondents. Some items have been adapted from previously validated instruments in the existing studies (Davis, 1989; Venkatesh et al., 2003; Slade et al., 2015; Jiang et al., 2019; Singh et al., 2020; Batucan et al., 2022; Nan et al., 2022). The corresponding statements were modified to suit the current research theme. Before disseminating the questionnaire, a pilot study on fifty local residents in China was conducted to check the context validity of the questionnaire statements, and the questionnaire was also reviewed by a group of experts.

### 3.2. Sampling and data collection

The refined questionnaire was disseminated online from October to November 2022 to residents from various regions of China. In this study, a combination of random sampling and convenience sampling was used throughout the sampling stage. The questionnaire was distributed to a number of online groups that were randomly selected from a list of public online chat groups. Respondents were asked if they were conveniently available to answer several questions regarding their behavioral intention to purchase the private pension scheme. They were also informed that their participation was entirely voluntary and that they could withdraw at any time during the process. At the end of the period, 462 valid responses were collected and used for further statistical analysis.

### 3.3. Data analysis

The survey data were first assessed by means of Cronbach’s alpha coefficients and exploratory factor analysis. The Cronbach’s alpha coefficient of a construct reflects the internal consistency of items, and it is a good measure of construct reliability. Exploratory factor analysis (EFA) can be used to identify the common factors that explain the structure and order of the measured items. The SPSS 26 statistical package is used to perform the calculation of Cronbach’s alpha coefficients and EFA. It is recommended that all variables are scored in the same direction (Streiner and Kottner, 2014). During the EFA process, scores on the items associated with perceived risk are therefore temporarily flipped to maintain the condition that all items are phrased in the same direction. Second, the validity of the outlined constructs was further assessed using confirmatory factor analysis (CFA). The convergent validity of the research model was reflected by the item loadings, composite reliability (CR) and average variance extracted (AVE). The discriminant validity was assessed based on the Fornell-Larcker criterion (Fornell and Larcker, 1981), which compares the square root of the AVE associated with a construct and its correlation with other constructs. Third, the SPSS AMOS 26 was used to perform structural equation modeling (SEM), and the analysis followed the recommendations from various sources (Hoyle, 1995; Yuan and Bentler, 2007; Kline, 2015). The coefficients of influencing paths in the research model were calculated to reflect the strength and the statistical significance of relationships. Mediation analysis was run *via* SEM through the method of bootstrapping.

### 3.4. Demographic profiles

The demographic profiles of the respondents are shown in Table 1. First, 51.30% of the respondents are female and 48.70% are male. Second, 37.01% of the respondents are 20–29 years old, 34.42% are 30–39 years old and the rest are 40–49 years old. It is also interesting to note that the 40–49 age group has the highest average score of 3.9223 on IP, while the 20–29 age group has the lowest. Third, a majority of the respondents have an annual income level of 50-200 k, while 36.36% have a level of 200-400 k and 14.50% have a level above 400 k. Fourth, the amounts of the respondents from four regions in China are close. It is noted that respondents from the South region and the East region have higher scores on IP than those from other regions. This might be attributed to the influence of pilot trials which China previously launched in Shanghai, Suzhou, and Fujian in 2018. Overall, there is not significant heterogeneity in terms of gender, age, income, and region. The sample size is also adequate for structural equation modeling (Kim, 2005; Iacobucci, 2010).

**Table 1:** Demographic profiles of the respondents (*n* = 462).

Characteristics		Frequency	Percentage (%)	Average IP score
Income	50 k ~ 200 k	227	49.13	3.8205
	200 k ~ 400 k	168	36.36	3.8720
	400 k+	67	14.50	3.8694
Age	20 ~ 29	171	37.01	3.7895
	30 ~ 39	159	34.42	3.8443
	40 ~ 49	132	28.57	3.9223
Gender	Female	237	51.30	3.8681
	Male	225	48.70	3.8233
Region	North	122	26.41	3.8074
	South	118	25.54	3.8814
	East	116	25.11	3.8966
	West	106	22.94	3.7972

## 4. Results

### 4.1. Cronbach’s alpha

To begin with, the Cronbach’s alpha values of constructs were calculated to reflect their reliability. According to the literature (Bland and Altman, 1997; Agbo, 2010; Taber, 2018), a Cronbach’s alpha value above 0.7 is considered acceptable. Table 2 shows that the Cronbach’s alpha values for all constructs are greater than 0.7, indicating that the items have good internal consistency. Besides, each corrected item-total correlation (CITC) value is greater than a threshold value of 0.5, and the corresponding Cronbach’s alpha if an item were deleted would be lower. Overall, the results suggest that all constructs are adequate, and items cannot be excluded from the constructs.

**Table 2:** The Cronbach’s alpha values.

Construct	Item	CITIC	Cronbach’s alpha if item deleted	Cronbach’s alpha
Time (TIME)	TIME1	0.809	0.855	0.901
	TIME2	0.821	0.843	
	TIME3	0.783	0.875	
Thought (THO)	THO1	0.647	0.741	0.806
	THO2	0.681	0.705	
	THO3	0.632	0.757	
Physical (PHY)	PHY1	0.682	0.751	0.823
	PHY2	0.687	0.746	
	PHY3	0.664	0.770	
Risk (RISK)	RISK1	0.605	0.767	0.800
	RISK2	0.685	0.683	
	RISK3	0.644	0.728	
Trust (TRUST)	TRUST1	0.600	0.715	0.777
	TRUST2	0.552	0.764	
	TRUST3	0.694	0.608	
Anticipation (ANT)	ANT1	0.688	0.773	0.829
	ANT2	0.696	0.765	
	ANT3	0.614	0.804	
	ANT4	0.636	0.794	
Social Influence (SI)	SI1	0.692	0.835	0.864
	SI2	0.721	0.823	
	SI3	0.759	0.807	
	SI4	0.682	0.840	
Effort Expectancy (EE)	EE1	0.773	0.873	0.900
	EE2	0.796	0.863	
	EE3	0.770	0.873	
	EE4	0.769	0.873	
Performance Expectancy (PE)	PE1	0.610	0.773	0.812
	PE2	0.644	0.757	
	PE3	0.565	0.797	
	PE4	0.709	0.727	
Side Benefits (SB)	SB1	0.706	0.838	0.869
	SB2	0.712	0.836	
	SB3	0.719	0.833	
	SB4	0.747	0.822	
Facilitating Conditions (FC)	FC1	0.727	0.861	0.884
	FC2	0.785	0.836	
	FC3	0.756	0.848	
	FC4	0.730	0.858	
Intention to Purchase (IP)	IP1	0.670	0.803	0.842
	IP2	0.633	0.818	
	IP3	0.681	0.798	
	IP4	0.724	0.778	

### 4.2. Exploratory factor analysis

The factorability of the data matrix is statistically reflected by a combination of Barlett’s test of sphericity (Bartlett, 1954) and the Kaiser-Meyer-Olkin test of sampling adequacy (Kaiser, 1974). The Bartlett’s test should produce a statistically significant chi-square value, while the KMO test should yield a value greater than 0.7 (Child, 2006; Hoelzle and Meyer, 2013). In this study, the Bartlett test returned a value of *p* less than 0.001 and the KMO test returned a value of 0.923, strongly supporting the application of EFA. Table 3 shows the results of the rotated component matrix with varimax rotation. First, twelve factors have been extracted, and each factor has an eigenvalue greater than 1. The extracted factors collectively explain 72.867% of the total variance. Second, the factor loadings in each component appear to match the anticipated structure of items. The items with factor loadings greater than 0.5 can be grouped into twelve distinct constructs, respectively. Overall, the EFA results suggest the soundness of the research model and the score validity.

**Table 3:** The exploratory factor analysis results (component matrix).

Indicator	Factor loading (rotated)											
	1	2	3	4	5	6	7	8	9	10	11	12
TIME1	0.165	0.121	0.119	0.132	0.068	0.081	0.101	**0.810**	0.228	0.097	0.117	0.118
TIME2	0.161	0.133	0.168	0.157	0.047	0.072	0.143	**0.815**	0.139	0.135	0.118	0.109
TIME3	0.152	0.103	0.154	0.115	0.132	0.119	0.119	**0.786**	0.149	0.102	0.196	0.058
THO1	0.201	0.055	0.125	0.232	0.038	0.125	0.096	0.195	0.093	**0.715**	0.097	0.084
THO2	0.151	0.054	0.112	0.120	0.061	0.055	0.141	0.107	0.177	**0.800**	0.097	0.004
THO3	0.189	0.041	0.133	0.027	0.076	0.077	0.150	0.030	0.054	**0.779**	0.000	0.181
PHY1	0.129	0.103	0.158	0.088	0.086	0.187	0.141	0.158	**0.741**	0.125	0.135	0.093
PHY2	0.138	0.117	0.135	0.066	0.117	0.045	0.210	0.118	**0.779**	0.073	0.067	0.128
PHY3	0.120	0.082	0.094	0.104	0.133	0.093	0.013	0.201	**0.767**	0.131	0.085	0.113
RISK1	0.097	0.095	0.050	0.067	0.100	0.122	0.206	0.206	−0.005	0.119	**0.735**	0.092
RISK2	0.081	0.096	0.125	0.149	0.101	0.255	0.079	0.140	0.115	0.082	**0.753**	0.119
RISK3	0.099	0.106	0.162	0.112	0.046	0.085	0.135	0.062	0.176	−0.007	**0.779**	0.114
TRUST1	0.137	0.040	0.043	0.070	0.122	0.086	0.072	0.039	0.247	0.096	0.159	**0.751**
TRUST2	0.007	0.011	0.154	0.137	0.112	0.158	0.096	0.226	−0.012	0.164	0.052	**0.711**
TRUST3	0.124	0.055	0.161	0.071	0.130	0.139	0.228	0.021	0.111	0.016	0.114	**0.790**
ANT1	0.020	0.153	0.121	0.120	**0.789**	0.021	0.109	0.049	0.102	0.080	0.052	0.052
ANT2	0.074	0.255	0.113	0.136	**0.778**	0.060	0.061	0.008	0.105	0.010	0.057	0.057
ANT3	−0.011	0.112	0.098	0.073	**0.732**	0.120	0.160	0.059	0.031	0.031	0.058	0.072
ANT4	0.089	0.075	0.060	0.070	**0.738**	0.160	0.120	0.102	0.082	0.054	0.067	0.160
SI1	0.097	0.113	0.069	**0.755**	0.156	0.096	0.108	0.114	0.092	0.108	0.122	0.050
SI2	0.066	0.027	0.221	**0.785**	0.092	0.045	0.190	0.022	0.110	0.033	0.116	0.064
SI3	0.069	0.079	0.152	**0.819**	0.086	0.133	0.117	0.091	0.035	0.097	0.030	0.072
SI4	0.083	0.166	0.108	**0.770**	0.084	0.091	0.026	0.136	0.026	0.102	0.056	0.083
EE1	**0.834**	0.074	0.082	0.109	−0.002	0.080	0.107	0.107	0.089	0.107	0.037	0.060
EE2	**0.840**	0.048	0.055	0.076	0.064	0.089	0.175	0.107	0.081	0.118	0.041	0.075
EE3	**0.800**	0.049	0.109	0.063	0.019	0.131	0.101	0.101	0.127	0.177	0.091	0.106
EE4	**0.822**	0.039	0.131	0.064	0.090	0.077	0.117	0.103	0.075	0.109	0.108	0.024
PE1	0.055	0.090	0.119	0.023	0.084	**0.740**	0.101	0.004	0.096	0.082	0.192	0.013
PE2	0.118	0.073	0.086	0.083	0.150	**0.758**	0.105	0.066	0.035	0.071	0.058	0.101
PE3	0.107	0.063	0.091	0.138	0.026	**0.701**	0.043	0.087	0.084	0.023	0.092	0.121
PE4	0.073	0.123	0.122	0.099	0.098	**0.781**	0.189	0.083	0.068	0.063	0.056	0.109
SB1	0.027	**0.799**	0.090	0.074	0.119	0.073	0.084	0.097	0.117	0.021	0.048	0.028
SB2	0.081	**0.780**	0.066	0.129	0.155	0.080	0.149	0.113	0.018	0.040	0.078	0.046
SB3	0.063	**0.798**	0.139	0.083	0.162	0.076	0.069	0.053	0.052	0.022	0.090	0.048
SB4	0.031	**0.837**	0.049	0.073	0.120	0.111	0.084	0.039	0.080	0.062	0.058	−0.014
FC1	0.122	0.100	**0.720**	0.170	0.118	0.119	0.133	0.168	0.115	0.187	0.054	0.134
FC2	0.107	0.122	**0.809**	0.149	0.118	0.089	0.097	0.088	0.123	0.100	0.084	0.127
FC3	0.118	0.132	**0.771**	0.173	0.113	0.135	0.101	0.115	0.062	0.139	0.174	0.003
FC4	0.077	0.060	**0.808**	0.110	0.103	0.144	0.119	0.081	0.103	0.009	0.059	0.111
IP1	0.145	0.083	0.121	0.125	0.124	0.165	**0.710**	0.020	0.104	0.186	0.103	0.086
IP2	0.133	0.140	0.114	0.116	0.070	0.154	**0.719**	0.128	0.081	0.095	0.024	0.102
IP3	0.158	0.075	0.117	0.108	0.189	0.060	**0.738**	0.093	0.102	0.049	0.171	0.106
IP4	0.135	0.171	0.104	0.132	0.164	0.129	**0.721**	0.133	0.094	0.115	0.181	0.126

### 4.3. Confirmatory factor analysis

Different from EFA, CFA can verify a pre-determined factor structure, and it is used to determine whether a model has sufficient efficiency and suitability for its purpose. As a statistical measure, CFA is adopted to identify whether scales are the ideal fit for data. In this paper, CFA was carried out with the help of SPSS AMOS 26. The measurement model results are tabulated in Table 4. The composite reliability (CR) values are calculated based on standard estimates, and they are all greater than a recommended cut-off value of 0.7 (Hoyle, 1995), while the corresponding calculated average variance extracted (AVE) are also greater than 0.5 (Fornell and Larcker, 1981), indicating the convergent validity of constructs. Second, Table 5 shows the model fit indices of the measurement model. Fitness indicators, such as Chi-square statistic (CMIN), CMIN/DF ratio, goodness-of-fit index (GFI), relative fit index (RFI), normed fit index (NFI), Tucker-Lewis index (TLI), comparative fit index (CFI) and root mean square error of approximation (RMSEA), are all above their recommended cut-off levels (Marsh et al., 2005; Singh, 2009). The model fit results clearly indicate the research model is suitable. Furthermore, the discriminant validity is also revealed by the results in Table 6. It is evident that the square root of the AVE associated with each construct is greater than its correlation coefficients with other constructs.

**Table 4:** The confirmatory factor analysis results.

Construct	Indicator	Test of parameters				Standardized estimate	Composite reliability	Convergent validity
		Estimate	S.E.	*t*-value	*p*		CR	AVE
Time	TIME3	1.000				0.842	0.902	0.754
	TIME2	1.094	0.047	23.356	***	0.889		
	TIME1	1.138	0.050	22.882	***	0.873		
Thought	THO3	1.000				0.714	0.806	0.581
	THO2	1.108	0.077	14.303	***	0.785		
	THO1	1.087	0.076	14.319	***	0.786		
Physical	PHY3	1.000				0.752	0.823	0.608
	PHY2	1.003	0.065	15.490	***	0.784		
	PHY1	1.029	0.065	15.748	***	0.803		
Risk	RISK3	1.000				0.742	0.802	0.576
	RISK2	1.104	0.073	15.183	***	0.827		
	RISK1	0.931	0.068	13.656	***	0.702		
Trust	TRUST3	1.000				0.845	0.786	0.553
	TRUST2	0.738	0.056	13.187	***	0.654		
	TRUST1	0.860	0.060	14.338	***	0.719		
Anticipation	ANT3	1.000				0.678	0.833	0.556
	ANT2	1.129	0.078	14.398	***	0.807		
	ANT1	0.986	0.070	14.120	***	0.783		
	ANT4	1.050	0.081	13.033	***	0.707		
Social influence	SI3	1.000				0.839	0.865	0.617
	SI2	0.928	0.049	19.001	***	0.805		
	SI1	0.820	0.047	17.551	***	0.754		
	SI4	0.878	0.051	17.131	***	0.740		
Effort expectancy	EE3	1.000				0.829	0.901	0.694
	EE2	0.997	0.046	21.496	***	0.855		
	EE1	0.875	0.043	20.522	***	0.826		
	EE4	0.937	0.046	20.333	***	0.821		
Performance expectancy	PE3	1.000				0.638	0.817	0.529
	PE2	1.119	0.088	12.657	***	0.743		
	PE1	0.980	0.082	12.014	***	0.691		
	PE4	1.180	0.088	13.419	***	0.825		
Side benefits	SB3	1.000				0.793	0.869	0.625
	SB2	0.950	0.055	17.339	***	0.784		
	SB1	0.927	0.055	16.991	***	0.770		
	SB4	0.996	0.055	18.045	***	0.814		
Facilitating conditions	FC3	1.000				0.818	0.886	0.660
	FC2	1.018	0.050	20.564	***	0.851		
	FC1	0.833	0.044	19.067	***	0.801		
	FC4	0.933	0.051	18.319	***	0.777		
Intention to purchase	IP3	1.000				0.750	0.843	0.573
	IP2	0.841	0.059	14.339	***	0.696		
	IP1	1.011	0.065	15.502	***	0.751		
	IP4	1.079	0.064	16.928	***	0.826		

S.E, Standard Error; CR, Composite Reliability; AVE, Average Variance Extracted; ***p < 0.001.

**Table 5:** The measurement model goodness-of-fit indices.

CMIN	CMIN/DF	GFI	RFI	NFI	TLI	CFI	RMSEA
	<3	>0.9	>0.9	>0.9	>0.9	>0.9	<0.08
918.577	1.157	0.917	0.906	0.917	0.986	0.988	0.018

**Table 6:** Discriminant validity of the model (implied correlation matrix).

	FC	SB	SI	ANT	TRUST	RISK	PHY	THO	TIME	PE	EE	IP
FC	0.812^a^											
SB	0.364	0.790^a^										
SI	0.499	0.339	0.786^a^									
ANT	0.414	0.484	0.394	0.746^a^								
TRUST	0.468	0.249	0.377	0.430	0.744^a^							
RISK	−0.472	−0.372	−0.429	−0.373	−0.477	0.759^a^						
PHY	0.483	0.358	0.372	0.414	0.504	−0.486	0.780^a^					
THO	0.478	0.254	0.440	0.293	0.419	−0.399	0.483	0.762^a^				
TIME	0.480	0.359	0.426	0.325	0.415	−0.519	0.568	0.479	0.868^a^			
PE	0.324	0.220	0.281	0.274	0.484	−0.535	0.340	0.281	0.327	0.728^a^		
EE	0.337	0.214	0.296	0.231	0.307	−0.316	0.438	0.525	0.446	0.215	0.833^a^	
IP	0.476	0.403	0.463	0.482	0.413	−0.437	0.424	0.406	0.412	0.440	0.457	0.757^a^

^a^ Square root of average variance extracted (AVE).

### 4.4. Path analysis

A set of hypothesized relationships between variables can be tested using structural equation modeling with the observed data. Path analysis is used to identify and evaluate the effects of a collection of factors operating on a specified outcome *via* different causal routes. The magnitude of a path coefficient represents the connecting strength of the effect, and the results of path analysis can help make inferences about the relative strength of causal relationships. According to Kline (1998), an adequate sample size in path analysis is recommended to be 10 times the number of factors. The ideal sample size for path analysis is 20 times the number of factors. The sample size in this study is considered sufficient.

First, the model fit indices of the structural model are present in Table 7. It has been previously shown in section 4.3 that a good-fitting measurement model is achieved, suggesting the model fits the observed data well and does not require respecification. As for the structural model, it is evident that CMIN, CMIN/DF, GFI, RMSEA and other indices are all acceptable, suggesting that the integrated FBM-UTAUT model is statistically reliable to predict the behavioral intention to purchase the private pension scheme.

**Table 7:** The structural model goodness-of-fit indices.

CMIN	CMIN/DF	GFI	RFI	NFI	TLI	CFI	RMSEA
	<3	>0.9	>0.9	>0.9	>0.9	>0.9	<0.08
980.023	1.205	0.912	0.902	0.911	0.982	0.984	0.021

Second, Table 8 shows the maximum likelihood estimates of the causal relationships. The SEM diagram is present in Appendix Figure A1. It can be seen that the proposed hypotheses were confirmed, with the contributions of factors from the ability dimension (EE and PE) being the most while the trigger dimension being the least. Venkatesh et al. (2003) also reported that EE and PE are among the strongest determinants of a person’s behavioral intention to use a technology. The three antecedent constructs of EE are all statistically significant with THO (*β* = 0.361, *p* < 0.001) being the most and PHY being the least (*β* = 0.161, *p* = 0.011). PE can also be significantly determined by RISK (*β* = −0.394, *p* < 0.001) and TRUST (*β* = 0.295, *p* < 0.001). It can be seen that the proposed antecedent frameworks can effectively explain the two critical variables in the ability dimension. Meanwhile, the UTAUT constructs, such as EE (*β* = 0.255, *p* < 0.001), PE (*β* = 0.226, *p* < 0.001), SI (*β* = 0.148, *p* = 0.007), and FC (*β* = 0.121, *p* = 0.031) have significant positive effects on IP at the 5% significance level. The results are in consistency with the majority of existing studies related to UTAUT (Williams et al., 2015; Dwivedi et al., 2020). It is also noted that SB (*β* = 0.107, *p* = 0.042) has a significant positive impact on IP. Furthermore, a bootstrap method was adopted to check the robustness of the estimates and the results are shown in Tables 9, 10. Table 9 presents the 95% percentile confidence intervals of the standard estimates, while Table 10 presents the 95% bias-corrected confidence intervals of the standard estimates. It is indicated that the bootstrapping standard estimates are all significantly consistent with slight variations of *p*-values.

**Table 8:** The maximum likelihood estimation of standard path coefficients.

Relationship	Standard *β*	S.E.	*t*-value	*p*	Decision
TIME ➔ EE	0.182	0.049	3.023	0.003	Supported
THO ➔ EE	0.361	0.068	5.883	***	Supported
PHY ➔ EE	0.161	0.055	2.533	0.011	Supported
RISK ➔ PE	−0.394	0.057	−6.054	***	Supported
TRUST ➔ PE	0.295	0.043	4.720	***	Supported
ANT ➔ IP	0.201	0.072	3.533	***	Supported
SI ➔ IP	0.148	0.074	2.714	0.007	Supported
SB ➔ IP	0.107	0.045	2.036	0.042	Supported
FC ➔ IP	0.121	0.057	2.162	0.031	Supported
EE ➔ IP	0.255	0.047	5.319	***	Supported
PE ➔ IP	0.226	0.067	4.494	***	Supported

***p < 0.001.

**Table 9:** The bootstrap estimation of standard path coefficients (95% percentile CI).

Relationship	Standard *β*	Lower bound	Upper bound	*p*	Decision
TIME ➔ EE	0.182	0.023	0.336	0.023	Supported
THO ➔ EE	0.361	0.208	0.502	0.001	Supported
PHY ➔ EE	0.161	0.016	0.309	0.031	Supported
RISK ➔ PE	−0.394	−0.540	−0.238	0.001	Supported
TRUST ➔ PE	0.295	0.155	0.444	0.001	Supported
ANT ➔ IP	0.201	0.069	0.330	0.001	Supported
SI ➔ IP	0.148	0.024	0.276	0.022	Supported
SB ➔ IP	0.107	−0.014	0.224	0.090	Supported
FC ➔ IP	0.121	−0.019	0.261	0.087	Supported
EE ➔ IP	0.255	0.138	0.374	0.001	Supported
PE ➔ IP	0.226	0.102	0.353	0.001	Supported

**Table 10:** The bootstrap estimation of standard path coefficients (95% bias-corrected CI).

Relationship	Standard *β*	Lower bound	Upper bound	*p*	Decision
TIME ➔ EE	0.182	0.023	0.336	0.023	Supported
THO ➔ EE	0.361	0.203	0.497	0.001	Supported
PHY ➔ EE	0.161	0.012	0.307	0.036	Supported
RISK ➔ PE	−0.394	−0.535	−0.233	0.001	Supported
TRUST ➔ PE	0.295	0.158	0.448	0.001	Supported
ANT ➔ IP	0.201	0.079	0.338	0.001	Supported
SI ➔ IP	0.148	0.030	0.282	0.017	Supported
SB ➔ IP	0.107	−0.010	0.228	0.082	Supported
FC ➔ IP	0.121	−0.015	0.263	0.081	Supported
EE ➔ IP	0.255	0.137	0.374	0.001	Supported
PE ➔ IP	0.226	0.107	0.358	0.001	Supported

Third, the mediating paths in the integrated FBM-UTAUT model were also confirmed with a bootstrap test for mediation. Specifically, the standard indirect effects and the corresponding 95% confidence intervals were calculated under both the percentile method and the bias-corrected method. The results are shown in Table 11, and the coefficients of connecting paths are also included. It can be seen that the mediating effects are all significant. Each calculated standard indirect effect is equal to the product value of two connecting path coefficients. Overall, it is evident that the intention of purchasing the private pension scheme can be effectively described using an integrated FBM-UTAUT model.

**Table 11:** The mediation analysis results.

Route	Standard indirect effect	95% percentile		95% bias-corrected	
		Lower	Upper	Lower	Upper
TIME ➔ EE ➔ IP	0.046	0.005	0.102	0.008	0.107
THO ➔ EE ➔ IP	0.092	0.039	0.160	0.042	0.163
PHY ➔ EE ➔ IP	0.041	0.004	0.089	0.005	0.091
RISK ➔ PE ➔ IP	−0.089	−0.161	−0.032	−1.660	−0.033
TRUST ➔ PE ➔ IP	0.067	0.024	0.125	0.027	0.134

## 5. Discussion

This research presents an integrated FBM-UTAUT model that can be used to predict a person’s intention to purchase the private pension scheme. First, the integrated FBM-UTAUT model was shown to be reliable in the current context, and it has a relatively good explanatory power as the classic UTAUT model (Venkatesh et al., 2003) or other adapted UTAUT models (Yee and Abdullah, 2021). All proposed relationships in the conceptual model have been tested to be significant, and the results regarding the coefficients of the UTAUT constructs (SI, EE, PE, and FC) were consistent with most previous studies (Williams et al., 2015; Khechine et al., 2016; Malik, 2020). We know from the literature that the UTAUT model has been widely used in various fields, such as information systems (Dwivedi et al., 2020), education (Aytekin et al., 2022), etc., to understand the factors that drive technology adoption and usage behavior. Digitization has become increasingly important in today’s digital age, as it enables organizations to store, manage, and access vast amounts of information quickly and easily. It has also revolutionized many industries, including entertainment, media, and finance, by allowing for the creation and distribution of digital content and the delivery of online services. With respect to the context of finance, in particular digital finance, the model has been used in several recent studies (Jadil et al., 2021; Firmansyah et al., 2023) to examine the adoption and usage. By using the UTAUT model, researchers and practitioners can gain insights into the key drivers of technology adoption and usage behavior in the financial industry and design strategies to increase the adoption and usage of financial technologies. We further show that the UTAUT model can be used in a pension context to understand behavioral intention. Meanwhile, the FBM model alone has been used in marketing campaigns to understand how to motivate consumers to take specific actions. But one of the drawbacks of the FBM is that it does not take external factors into consideration, such as the influence of friends and family, facilitating conditions. By combining the FBM and the UTAUT model, we could gain a deeper understanding of the underlying factors that influence technology usage and develop more effective strategies to encourage and support desired behaviors.

Second, the research findings reveal that, among the six critical factors (ANT, SI, EE, PE, SB, and FC) directly impacting purchase intention, EE is the most influential factor with a standard coefficient of 0.255, and PE is the second most influential factor with a standard coefficient of 0.226. The results are in line with most recent studies in similar contexts (Chang et al., 2022; Jena, 2022; Nan et al., 2022). Most existing studies in other contexts also generally support the significant effects of EE and PE on behavioral intention (BI). The relative influence of effort expectancy and performance expectancy on technology adoption and usage can vary depending on the specific context and individual. In general, performance expectancy is often considered to be a stronger predictor, as individuals are more likely to adopt and use technology that they believe will improve their performance and meet their goals. Studies have also shown that performance expectancy can also have a positive influence on effort expectancy (Sung et al., 2015; Fedorko et al., 2021; Li et al., 2023), as individuals may be more willing to put in the effort to learn and use a technology if they believe it will have a positive impact on their performance. However, effort expectancy can also play an important role. People are less likely to adopt and use technology that requires a high level of effort, such as a complex or difficult-to-use interface, or that requires extensive training or support. If the technology is perceived as being too difficult or time-consuming to learn and use, individuals may be less likely to adopt it, regardless of its potential performance benefits. With respect to the private pension scheme, it could be more difficult to understand its account rules and clauses than other financial products. However, the effects of SI and FC on BI can vary depending on the specific context. For example, Li et al. (2023) reported a statistically nonsignificant relationship between FC and BI in a remote health management service context and Batucan et al. (2022) found that SI has no significant impact on BI in an online learning system context.

Third, the determinants of EE and PE are all shown to be significant, and their indirect effects on purchase intention are also statistically robust. This further helps verify the suitability of the integrated FBM-UTAUT model in the current context. In this study, we specifically show that EE and PE can be influenced by a number of antecedents, including time effort, thought effort, physical effort, risk, and trust. The calculated squared multiple correlations for EE and PE are 0.341 and 0.354, respectively, suggesting the explanatory power of the proposed antecedents is acceptable. Understanding the determinants of EE and PE can further help develop pension products that are easy to purchase and provide clear performance benefits, thus promoting the coverage of the private pension scheme.

This research also elicits several important implications for financial institutions and policymakers. First, under the private pension scheme, people will have more options to invest their pensions in various financial products, and the scheme system provides a lucrative market for insurance companies, banks and many other financial institutions. The financial industry is therefore recommended to embrace this opportunity and optimize the design and delivery of pension products accordingly. For example, financial companies can upgrade their service infrastructures, streamline the purchasing process, fully disclose relevant product information, reduce management fees, and improve customer service quality. These actions would undoubtedly help improve people’s expectations and beliefs about performance, ease of purchase, product risk, benefits, and the trustworthiness of the scheme. Since real estate has historically accounted for the majority of private investment in China, many residents may be unfamiliar with the concept of pension investments. Building consumer trust and enhancing investor awareness and education will thus be crucial measures for businesses looking to break into the market. Second, in order to improve the number of enrolled residents with respect to the private pension scheme, policymakers hope to know what behavioral influences should be taken into account. This study has tested an integrated FBM-UTAUT model to explain purchase intention. The behavioral elements discussed are those that might either hinder or help purchase intention and may be considered in future policy solutions. In order to guarantee the public’s active involvement in the program, sound policy guidelines and guidance will be needed. To promote the use of private pensions, tax incentive policies, product regulations, customer protection policies, and information campaigns, will be crucial. The governmental authorities are also obliged to offer additional clarification and direction on activities that are permitted and encouraged.

## 6. Conclusion

This study has explored critical factors affecting the intention to purchase China’s private pension scheme based on an integrated FBM-UTAUT model. The integrated FBM-UTAUT model consists of six direct factors in motivation dimension, ability dimension, and trigger dimension, respectively, and it also incorporates the key determinants of effort expectancy and performance expectancy. The survey-based data were collected and then analyzed through exploratory factor analysis, confirmatory factor analysis, and path analysis. Essentially, the integrated FBM-UTAUT exhibits an explanation of more than 70% of total variance and all six direct factors, including anticipation, social influence, effort expectancy, performance expectancy, side benefits, and facilitating conditions, were found to have significant positive impacts on intention to purchase. Furthermore, the determinants of effort expectancy and performance expectancy were shown to be effective, and their indirect effects on intention to purchase were also confirmed. The findings support the use of the integrated FBM-UTAUT model to explain purchase intentions in the context of a private pension scheme. Financial institutions and policymakers could find the current research findings helpful for designing improved pension products and issuing well-rounded pension policies.

Although the current research has obtained important results and elicited some meaningful implications, it still has several limitations. First, the study only assessed people’s intention to purchase private pension scheme quantitatively, it is recommended to carry out some extra qualitative research, such as interviews and focus group discussions, to shed more light on people’s attitudes and beliefs toward the scheme. This also helps identify any potentially significant omitted factors. Second, the present study does not analyze some potential moderating variables, such as gender, age, education, income, financial literacy, etc. These moderating variables may help explain why some individuals purchase the scheme more readily than others. By considering the moderating variables, the generalizability of the integrated model could be enhanced. Third, the integrated model has been shown to be reliable in the current context, and more empirical evidences are needed to test its adaptability in similar contexts.

Researchers are also recommended to consider the following research directions. First, it could be academically interesting to apply the model in different countries. The pension system in a specific country plays a critical role in ensuring its retirement security, promoting economic growth, fostering intergenerational fairness, maintaining social stability, and supporting inclusive growth. Besides, researchers could explore how cultural and diversity variables influence the intention to purchase. Second, the UTAUT model incorporates several predictor variables, but new technology developments and advancements may warrant the consideration of additional new variables. Third, it is also interesting to explore the integration of the current model with other classic models, to expand the understanding of behavioral intention.

## Data availability statement

The raw data supporting the conclusions of this article will be made available by the authors, without undue reservation.

## Ethics statement

The studies involving human participants were reviewed and approved by the Sanda University Ethics Committee. Written informed consent for participation was not required for this study in accordance with the national legislation and the institutional requirements. Written informed consent was obtained from the individual(s) for the publication of any potentially identifiable images or data included in this article.

## Author contributions

GW and JG: conceptualization, methodology, data collection, software, data analysis, manuscript preparation, and project management. GW: investigation, formal analysis, review, editing, supervision, and funding arrangement. All authors have read and agreed to the published version of the manuscript.

## Funding

This research was support by Science and Technology Commission of Shanghai Municipality (No. 22692195000), Shanghai Planning Office of Philosophy and Social Science (No. 2021ZJB005), and the Sanda University Research Fund (Nos. 2021ZD03 and 2022YB18).

## Conflict of interest

The authors declare that the research was conducted in the absence of any commercial or financial relationships that could be construed as a potential conflict of interest.

## Publisher’s note

All claims expressed in this article are solely those of the authors and do not necessarily represent those of their affiliated organizations, or those of the publisher, the editors and the reviewers. Any product that may be evaluated in this article, or claim that may be made by its manufacturer, is not guaranteed or endorsed by the publisher.

## Supplementary material

The Supplementary material for this article can be found online at: https://www.frontiersin.org/articles/10.3389/fpsyg.2023.1136351/full#supplementary-material

SUPPLEMENTARY TABLE A1 The items of questionnaire.

SUPPLEMENTARY FIGURE A1 The SEM diagram.
