# Supplementary material for: Investigating the intention of purchasing private pension scheme based on an integrated FBM-UTAUT model: The case of China
Source: Front Psychol. 2023 Mar 9;14:1136351. doi: 10.3389/fpsyg.2023.1136351 (PMC10033583; doi:10.3389/fpsyg.2023.1136351)

Appendix

TABLE A1. The items of questionnaire

Constructs	Items	Source
Anticipation	ANT1. I have a positive expectation about the private pension scheme.	Self-developed
	ANT2. I expect that I can maintain my standard of living after I retire.	Self-developed
	ANT3. I hope the pension fund is inflation-hedged.	Self-developed
	ANT4. I hope that the pension scheme provides various benefits.	Self-developed
Social Influence	SI1. I think that my family and friends would support the purchase.	(Venkatesh et al., 2003)
	SI2. I expect myself to be more likely to purchase if my friends purchase it.	(Slade et al., 2015)
	SI3. People who influence my behavior think that I should purchase the private pension scheme.	Self-developed
	SI4. I might want to know how my friends feel about it before I purchase it	(Singh et al., 2022)
Effort Expectancy	EE1. I think that purchasing the private pension scheme is easy.	(Venkatesh et al., 2003)
	EE2. I think it does not take too much efforts to purchase the private pension scheme.	(Jiang et al., 2019)
	EE3. It would be easy for me to complete the purchasing.	(Nan et al., 2022)
	EE4. I would find the private pension scheme easy to understand.	(Venkatesh et al., 2003)
Time	TIME1. I think that it does not take too much time to purchase the private pension scheme.	Self-developed
	TIME2. I think that little time is required to comprehend the scheme.	Self-developed
	TIME3. I think that tracking my pension account balance can be quick.	Self-developed
Thought	THO1. I think that understanding the pension scheme is not difficult	Self-developed
	THO2. I think that I am able to digest relevant information easily	Self-developed
	THO3. I think that it does not require some expertise to purchase the private pension scheme.	Self-developed
Physical	PHY1. I think that purchasing the private pension scheme does not require too much physical efforts.	Self-developed
	PHY2. I think that checking my pension account on a daily basis is not physically troublesome.	Self-developed
	PHY3. I think that purchasing the scheme requires minimal physical effort.	Self-developed
Performance Expectancy	PE1. I think that the private pension scheme would financially help improve my retirement life.	(Venkatesh et al., 2003)
	PE2. I think that my net worth after retirement can be higher if I purchase the private pension scheme.	Self-developed
	PE3. I think that the private pension scheme manager has excellence in investment.	(Jiang et al., 2019)
	PE4. I think that the private pension scheme is less risky than other investment vehicles.	(Slade et al., 2015)
Risk	PR1. I think that the private pension scheme has a high risk.	(Nan et al. 2022)
	PR2. I reckon that the pension management company has a high risk of default.	Self-developed
	PR3. I think the possibility of losing my money in the pension account is high.	Self-developed
Trust	TR1. I think the pension management company is trustworthy.	(Slade et al., 2015)
	TR2. I trust the professionalism of the pension management company.	Self-developed
	TR3. I think that all parties would behave in line with the pension contracts.	Self-developed
Side Benefits	SB1. I think that buying this product could bring me additional benefits	Self-developed
	SB2. I think that searching for other benefits like discounts or giveaways is important.	Self-developed
	SB3. I will pay more attention to the long-term benefits this product could bring to me.	Self-developed
	SB4. I think that the tax benefit of this product can be very critical.	Self-developed
Facilitating Conditions	FC1. I think that I have the resources necessary to purchase the private pension scheme.	(Venkatesh et al., 2003)
	FC2. I think that I have the knowledge necessary to purchase the private pension scheme.	(Slade et al., 2015)
	FC3. I think that support is available when I have questions regarding the private pension scheme.	Self-developed
	FC4. I think that our country has support (infrastructures, policies, etc.) for the private pension scheme.	(Batucan et al., 2022)
Intention to Purchase	IP1. I am willing to purchase the private pension scheme.	Self-developed
	IP2. I will recommend my family members to purchase the private pension scheme.	(Davis, 1989)

	IP3. I will recommend my friends to purchase the private pension scheme.	(Nan et al., 2022)
	IP4. I think that I am inclined to purchase the private pension scheme as soon as possible.	Self-developed

FIGURE A1. The SEM diagram

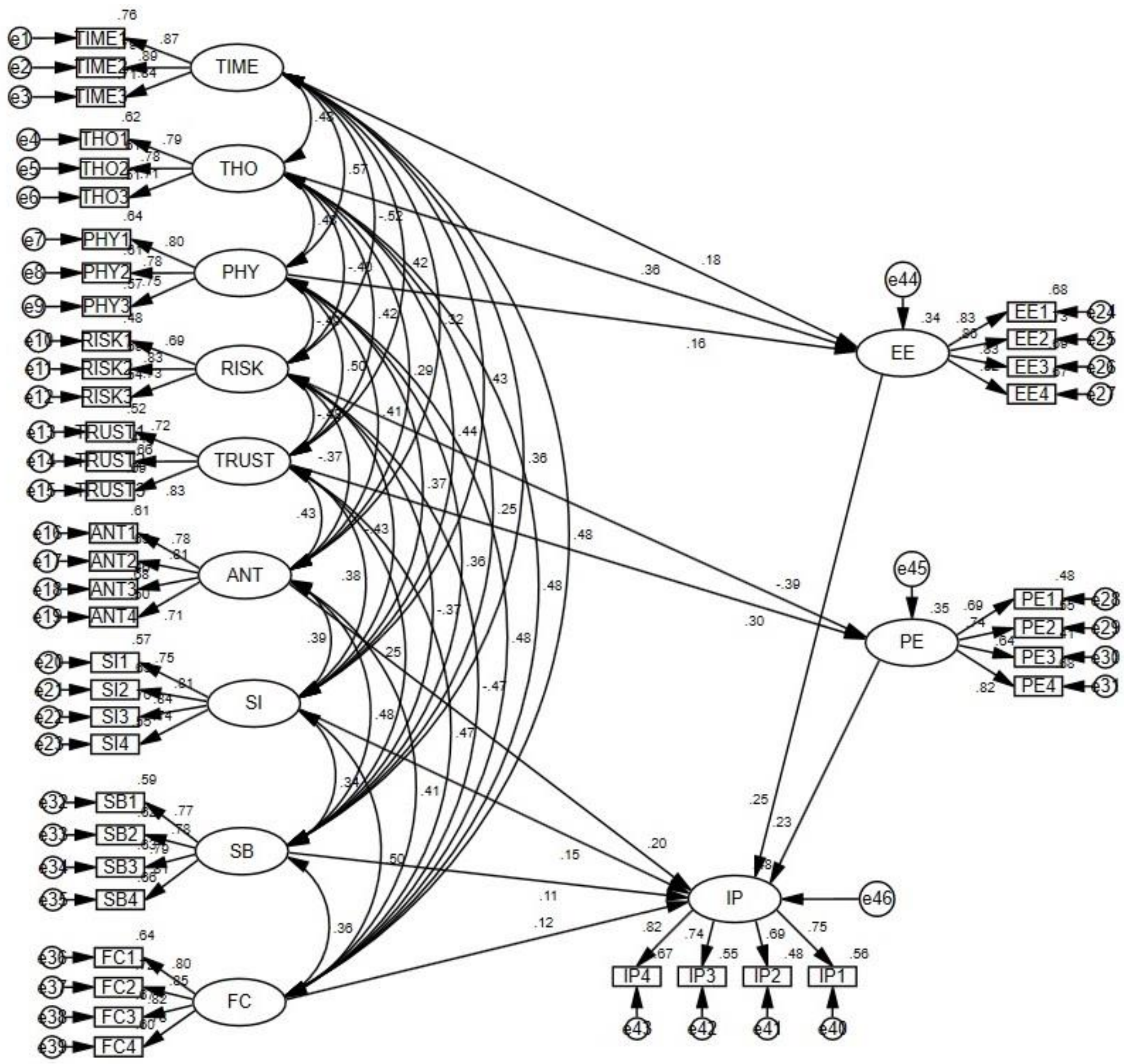

Supplement: SUPPLEMENTARY TABLE A1 — The items of questionnaire. [file Data_Sheet_1.pdf]
